# Implementation of a fully virtual enterprise-wide clinical evidence-based suicide prevention program in the U. S. Department of Veterans Affairs: the suicide prevention 2.0 clinical telehealth initiative

**DOI:** 10.3389/fpsyt.2025.1668417

**Published:** 2026-02-10

**Authors:** Sara J. Landes, Jessica A. Walker, Nicole M. Bekman, Mandy J. Kumpula, Samantha L. Lhermitte, Rani A. Hoff, Lisanne M. van Engelen, Lisa M. Betthauser, Sherry A. Beaudreau, Wendy H. Batdorf, Lisa K. Kearney, Matthew A. Miller, Jeffery A. Pitcock, Meaghan A. Stacy

**Affiliations:** 1 Behavioral Health Quality Enhancement Research Initiative (QUERI), Central Arkansas Veterans Healthcare System, North Little Rock, AR, United States; 2 Department of Psychiatry, University of Arkansas for Medical Sciences, Little Rock, AR, United States; 3 Office of Suicide Prevention, VA Central Office, U. S. Department of Veterans Affairs, Washington DC, United States; 4 Women’s Mental Health, Office of Mental Health, U.S. Department of Veterans Affairs, Washington DC, United States; 5 Center for Clinical Management Research, VA Ann Arbor Healthcare System, Ann Arbor, MI, United States; 6 Northeast Program Evaluation Center, Office of Mental Health, Veterans Health Administration U. S. Department of Veterans Affairs, West Haven, CT, United States; 7 Department of Psychiatry, Yale University School of Medicine, New Haven, CT, United States; 8 Rocky Mountain Mental Illness Research Education and Clinical Center (MIRECC), U. S. Department of Veterans Affairs, Aurora, CO, United States; 9 Sierra Pacific Mental Illness Research Education and Clinical Center (MIRECC), VA Palo Alto Health Care System, Palo Alto, CA, United States; 10 Department of Psychiatry and Behavioral Sciences, Stanford University School of Medicine, Stanford, CA, United States; 11 School of Psychology, University of Queensland, Brisbane, QLD, Australia; 12 National Evidence-Based Psychotherapy, Office of Mental Health, U.S. Department of Veterans Affairs, Washington DC, United States; 13 VA Central Office, U. S. Department of Veterans Affairs, Washington DC, United States; 14 Department of Psychiatry, University of Texas Health - San Antonio, San Antonio, TX, United States

**Keywords:** suicide prevention, suicidality, veterans, telehealth, implementation, evidence-based psychotherapy

## Abstract

**Introduction:**

Veteran death by suicide is a complex issue made up of many factors. Despite the high need for mental health treatment, and treatments that specifically target suicide, evidence-based psychotherapies (EBPs) are difficult to access, even more so in rural areas. In concordance with the 2018 National Strategy for Preventing Veteran Suicide, VA suicide prevention leadership developed Suicide Prevention 2.0 (SP 2.0) to implement a public health model that includes community-based prevention strategies and improves clinical interventions within VA. The Suicide Prevention 2.0 Clinical Telehealth program was implemented in each of VA’s 18 regional Clinical Resource Hubs and expanded clinical intervention strategies within VA by implementing four EBPs for Suicide Prevention (EBP-SP) via telehealth: the Safety Planning Intervention, Problem-Solving Therapy for Suicide Prevention, Cognitive Behavioral Therapy for Suicide Prevention, and Dialectical Behavior Therapy.

**Methods:**

A wide variety of implementation strategies were used (e.g., access new funding, training, consultation, create new clinical teams). The primary inclusion criterion for veteran referral to SP 2.0 Clinical Telehealth is a recent history of suicidal self-directed violence. Implementation was guided by the Exploration, Preparation, Implementation, and Sustainment (EPIS) framework and RE-AIM was used as an evaluation framework.

**Results:**

By April 2023, SP 2.0 Clinical Telehealth services were available in all 18 regions and in 139 of 139 (100%) VA health care systems in the U.S. By the end of September 2024, the program had hired 137 therapists and retained 78.10% in their role, and 100% were trained in two or more EBP-SPs. By the end of September 2024, the program received 23,628 referrals nationwide. Increasing referral rates year over year suggests ongoing sustained reach.

**Discussion:**

SP 2.0 Clinical Telehealth represents the first and only enterprise-wide fully virtual evidence-based treatment program for veterans with a recent history of suicidal self-directed violence. The program’s implementation was successful in reaching all VISNs and all VA health care systems in the U.S. The SP 2.0 Clinical Telehealth program can be used as a model for other large health care systems looking to improve provision of evidence-based interventions for suicide prevention.

## Introduction

### Suicide is a public health crisis

Suicide is an urgent public health crisis, with rates on the rise since 2000 (1). Suicide is an even greater issue for veterans. In 2022, the age-adjusted suicide rate for male and female veterans was 44% and 92% greater than that of non-veteran male and female adults respectively (2). In 2022, suicide was the 12th-leading cause of death for veterans overall and the second-leading cause of death among veterans age 45 and younger (3). That same year, 6,407 veterans died by suicide (2). Veteran death by suicide is a complex issue made up of many factors, and this tragic loss of life cannot be solved by a single intervention or initiative (4). Mental health and substance use disorder diagnoses put veterans at an increased risk for suicide (5), along with other societal issues such as housing insecurity and legal system involvement (6, 7).

### Difficulty getting treatment for mental health and suicide

Despite the high need for mental health treatment, and treatments that specifically target suicide, evidence-based psychotherapies (EBPs) are difficult to access. In general, it is well documented that even though EBPs exist, most therapists do not receive training in or use EBPs in routine practice (8–11). Access is even more limited for those who live in rural and/or medically underserved areas (12, 13).

### The critical importance of expanding access to EBPs

To address these issues for veterans, in 2006 the U.S. Department of Veterans Affairs (VA) initiated an organization-wide dissemination and implementation effort to train therapists in EBPs for posttraumatic stress disorder (PTSD) (14). In 2008, this was followed by the development of an Evidence-Based Psychotherapy Program to disseminate and train therapists in additional EBPs nationally (14, 15). As such, the EBP Program has a longstanding history of successful competency-based training in EBPs, which involves multi-component didactic and experiential training followed by structured case consultation (15). This model of training in EBPs has demonstrated significant, positive therapist training outcomes, including increased clinical competencies (16–18), enhanced self-efficacy (19), and improved knowledge and attitudes (20). VA EBP program evaluation results also indicate that veterans served by EBP-trained therapists demonstrate symptom reductions with effect sizes in the medium-to-large or large range among primary patient outcomes, as well as significant improvements in quality of life (16, 18–23). VA has supported national dissemination of EBP training in interventions that address PTSD, major depressive disorder, substance use disorders, insomnia, chronic pain, serious mental illness, motivation and engagement, and relationship distress. Since 2007, more than 15,000 unique therapists have been trained to competency in one or more EBPs via national training and dissemination efforts (24).

As an early adopter of system-wide implementation methods, VA’s traditional EBP training program model has demonstrated effectiveness in achieving therapist competency to deliver EBPs and improving known provider-level facilitators of implementation. Despite VA’s successes, barriers to sustained adoption and implementation of the interventions post-training include staff turnover, scheduling difficulties, patient volume, clinician workload (25), inconsistent institutional support, and lack of an EBP-focused clinical mission (26). Of note, these barriers are not unique to VA and are common across settings implementing EBPs (27–29). Because of the significant variability in these factors across VA medical centers, ensuring national access to an EBP with the VA’s existing dissemination and training models has been a challenge, resulting in substantial variability in rates of veteran reach and therapist adoption between VA facilities and clinics (25, 30). As a learning healthcare system, VA has used a variety of strategies to improve implementation outcomes, including EBP Coordinators to serve as internal facilitators at VA facilities, national policy requiring EBP availability, and performance metrics and monitoring systems to support EBP tracking and accountability (15). Rates of EBP implementation are relatively low in many regions and facilities across VA (31). EBP adoption and reach tend to be highest in specialty mental health settings where therapists experience fewer competing time demands, have frequent access to veterans with the target condition(s) for the EBP, and have consistent leadership and structural supports to deliver EBPs (32). VA continues to use this information to improve upon methods by which to implement EBPs.

### Implementation science can aid program implementation

Implementation science is the “scientific study of methods to promote the systematic uptake of research findings and other evidence-based practices into routine practice, and, hence, to improve the quality and effectiveness of health services.” (33) As described above, there are challenges to implementing EBPs into routine care. Establishing the effectiveness of an intervention does not guarantee its uptake into routine care (34). Within the field of implementation science there are a variety of theories, models, and frameworks to guide both implementation planning and evaluation (35). The field has also specified implementation strategies to “enhance the adoption, implementation, and sustainability of a clinical program or practice.” (36) Use of a theory, model, or framework and implementation strategies that target barriers and facilitators to implementation can enhance the likelihood that an intervention is successfully implemented (37). VA has a long history of both supporting and benefiting from implementation research (38–42), as operational leaders within VA frequently partner with implementation science experts.

### Current paper

In 2018, the VA released a new national strategy for preventing veteran suicide that called for both enhancing care for individuals at risk within the VA health care system and adding a public health approach to reach all veterans, both those inside and outside of the VA system (43). To move forward a full public health approach to veteran suicide prevention, VA suicide prevention leadership developed Suicide Prevention 2.0 (SP 2.0) to implement community-based prevention strategies, embarking to reach all veterans, while simultaneously ensuring expansion of evidence-based clinical strategies (4, 44). This work built on VA’s experience and knowledge of implementing EBPs and used implementation science strategies to ensure program implementation success. The goal of the present manuscript is to describe the implementation of the Suicide Prevention 2.0 Clinical Telehealth program and report on initial reach, adoption, and maintenance outcomes.

## Methods

### Setting

The Veterans Healthcare Administration (VHA) is the arm of VA that provides health care to veterans; it is the largest integrated health care system in the U.S. VHA is comprised of 18 regional networks, referred to as Veteran Integrated Service Networks (VISNs). Care is provided at 1,380 health care facilities that include 170 medical centers and 1,193 outpatient sites. Over 9 million veterans are enrolled in VHA care. VHA facilities offer a range of health and mental health services (https://www.va.gov/health/aboutvha.asp). Each VISN is home to a VISN Clinical Resource Hub (CRH), which provides a telehealth model of care (45). Every CRH offers virtual primary care and mental health services and may offer specialty care services based on the needs of their VISN (e.g., dermatology, sleep medicine, etc.). Methods and data ascertainment for analyses were considered non-research and did not require institutional review board (IRB) approval per the Department of Veterans Affairs Office of Research and Development Program Guide 1200.21. In addition, the VA Connecticut Healthcare System Research Department designated this as a non-research quality improvement project and confirmed IRB review was not required.

### Implementation framework

Implementation and evaluation were guided by the Exploration, Preparation, Implementation, and Sustainment (EPIS) (46, 47) framework. The EPIS framework was developed based on implementation science literature in public sector social and allied health service systems. EPIS defines four phases that align with the implementation process:

1. Exploration (awareness of health need to be addressed, identification of practices to be implemented),
2. Preparation (selection of practice(s) to implement, assessment of barriers and facilitators to implementation, development of an implementation plan, selection of implementation strategies),
3. Implementation (practice use is initiated, monitoring of implementation process, adjusting strategies as needed) and,
4. Sustainment (structures, processes, and supports are ongoing so the practice continues being offered to realize an impact).

**Figure 1:**
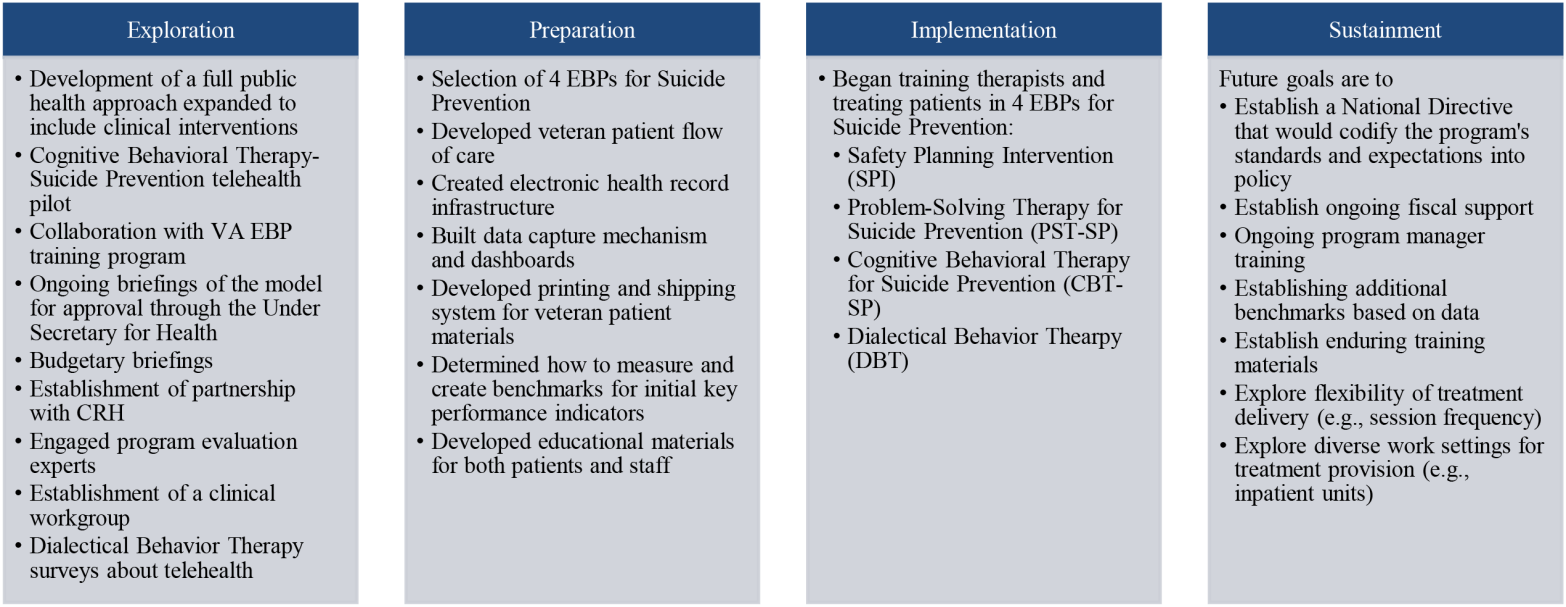
Select activities that occurred during each EPIS phase.

**Figure 2:**
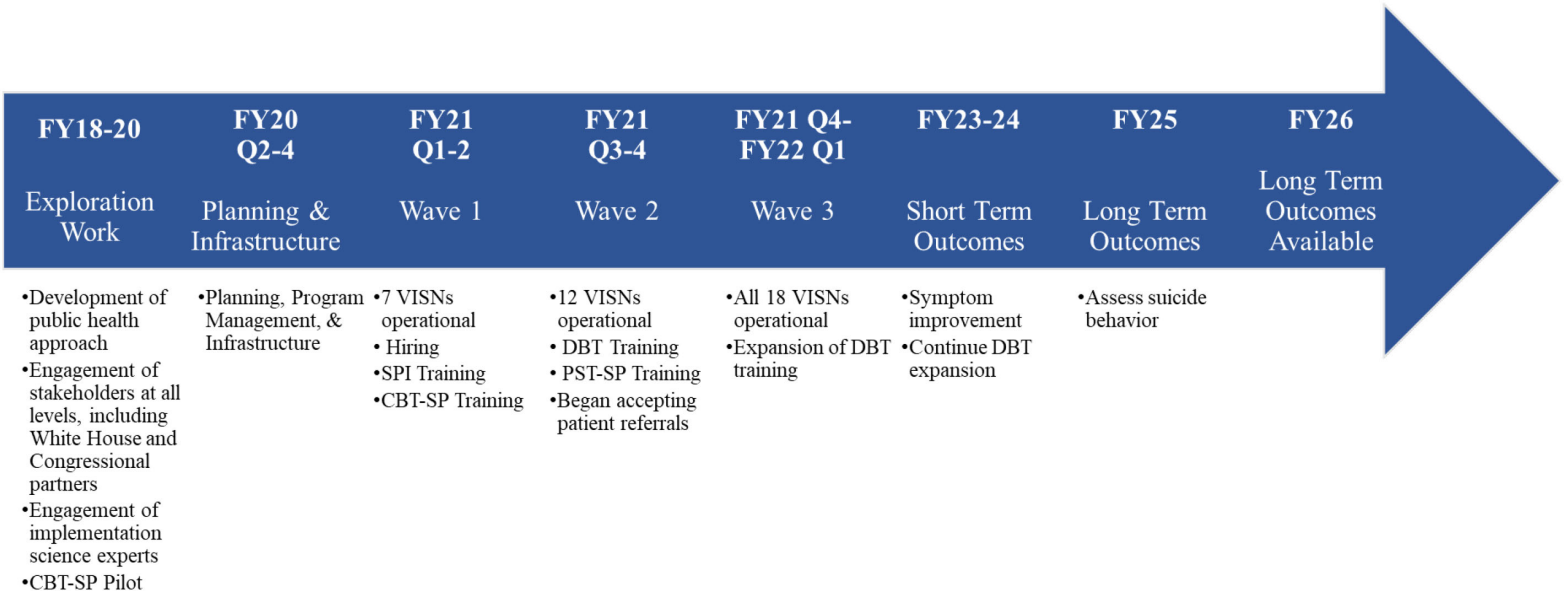
Overall timeline of implementation activities.

In addition to phases of the implementation process, EPIS defines constructs that impact implementation, including outer context, inner context, innovation factors (e.g., characteristics of the EBP), and bridging factors (i.e., interconnections between these constructs) (47). More detail can be found at www.episframework.com. See **Figure 1** for activities that occurred during each phase of implementation and **Figure 2** for an overall timeline of implementation activities that also includes when different outcomes will become available.

### Context

VA suicide prevention leaders engaged stakeholders across the VA system to develop SP 2.0. Stakeholders included medical center directors, VISN Network Directors, VISN Chief Mental Health Officers (CMHOs), CRH leadership, and different program offices like the Office of Primary Care. Initial engagement with stakeholders also included briefings with external partners such as Veteran Service Organizations, Congressional staff, and the White House to respond to their questions and obtain their input on implementation of a full public health approach to suicide prevention. This stakeholder engagement (48) was needed for both program design and the culture shift needed at senior levels of leadership to agree to such an innovative approach to suicide prevention that included engaging community partners to reach veterans not enrolled in VA health care.

As the community-based prevention portion of SP 2.0 moved through the initial approval processes, VA suicide prevention leaders also engaged implementation scientists and suicide prevention national research experts in identifying the most promising, evidence-based clinical approaches to include in the SP 2.0 model. The release of the 2019 revised clinical practice guideline (CPG) for assessment and management of patients at risk for suicide occurred during the development of SP 2.0, which served as the foundation of evidence to inform the decision making on which clinical treatments to promote in this new initiative (49). Given the limited availability of EBPs (15, 50) and the release of a new guideline, SP 2.0 provided an opportunity to expand clinical intervention strategies within VA by identifying EBPs with the best outcomes to expand nationwide access.

Those EBPs for Suicide Prevention (EBP-SP; described in detail below) included the Safety Planning Intervention (SPI), Problem-Solving Therapy for Suicide Prevention (PST-SP), Cognitive Behavioral Therapy for Suicide Prevention (CBT-SP), and Dialectical Behavior Therapy (DBT). However, at that time, there were limited VA therapists trained in these EBP-SPs and there was concern about how to ensure veteran access across the country to trained providers. Program development efforts addressed these barriers by obtaining a financial commitment to fund therapists whose time would be fully dedicated to deployment of EBP-SPs and to develop EBP-SP training programs. In addition, leadership decided to deliver these EBP-SPs using a telehealth delivery method to maximize veteran access across the nation. VA suicide prevention leaders met with the VA’s EBP program to learn from their experience in training VA therapists, which highlighted the importance of competency-based training in EBP. Collaborative work was also done with VA’s CRHs to establish a partnership such that SP 2.0 Clinical therapists and supervisors would work within existing VISN-based CRH infrastructure. Collaborations with CRH included creating a framework for patient flow, building clinics specifically for telehealth, and partnering with the Office of Connected Care (VA’s office that brings VA digital technology to veterans and health care professionals) for utilization of the “digital divide” consult to connect veterans to internet capable devices, therefore bridging the digital divide. CRH partnership also supported and facilitated relationship building with local facility leaders and stakeholders. SP 2.0 Clinical established standards for therapists, particularly to protect their time for focused delivery of EBP-SPs, a lesson learned from prior EBP implementation. VA suicide prevention leadership also engaged additional experts in suicide prevention and program evaluation to plan the program, its implementation, and its evaluation.

VA suicide prevention leadership worked through different levels of governance, resolved the financial details with VHA finance, and obtained final approval for SP 2.0 from the Under Secretary for Health. After approval was obtained, suicide prevention leadership created workgroups that developed implementation and evaluation plans. Two work groups were established in March 2020: 1) the SP 2.0 Clinically-Based Interventions Work Group and 2) the Evaluation and Implementation Work Group. The work groups were tasked with developing logic models, identifying project outcomes to assess, creating an implementation and data collection plan, and developing an implementation checklist.

To inform SP 2.0 Clinical Telehealth Initiative program development, a telehealth CBT-SP program pilot was conducted in two VISNs from 2019 to 2021. The pilot trained 10 therapists as CBT-SP experts and developed and implemented substantial programmatic infrastructure, including: a telehealth CBT-SP therapy manual with incorporated clinical measures (e.g., session check-in questions regarding suicidal ideation and behaviors, standardized measures of depression and suicide), a patient therapy workbook, referral and care documentation templates, etc. The pilot accepted new veteran patients between February 2019 and March 2021, at which time the program was subsumed under the SP 2.0 Clinical Telehealth initiative. There were 436 veterans referred to the pilot, 223 (51.14%) of whom completed at least one CBT-SP therapy session. Of those 223 who initiated CBT-SP therapy, 140 (62.78%) completed a full treatment course (12–14 sessions). Manual electronic health record (EHR) abstraction and data analysis found no significant demographic or mental health diagnostic differences between those who refused CBT-SP, those who left treatment early, and those who received a full course of treatment. Veterans who received at least 1 session of CBT-SP reported a significant improvement in depression as measured by the Patient Health Questionnaire (PHQ-9) (51), with those receiving a full course of CBT-SP reporting a larger reduction in symptoms. Across all levels of participation, there were significant reductions in self-reported suicidal ideation and suicidal behavior. Findings generally supported the feasibility and acceptability of this telehealth program model. Ultimately, this pilot provided proof of concept and information regarding implementation strategies, logistics, potential barriers and facilitators, and best practices for a national initiative. Of note, because the CBT-SP pilot and SP 2.0 Clinical Telehealth initiative were based on a virtual model, program implementation was well-suited to navigate the uncertainties of the COVID-19 pandemic that began midway through the CBT-SP pilot.

The SP 2.0 Clinical Telehealth Program was conceptualized to maximize facilitators of EBP implementation while reducing implementation barriers common within the traditional mental health continuum of care in VHA. See **Table 1** for challenges of the traditional facility-based EBP training program model and how the SP 2.0 Clinical Telehealth initiative was designed to address those challenges.

**Table 1:** Challenges of the traditional facility-based EBP training program model and how those challenges are addressed in the SP 2.0 Clinical Telehealth model.

Challenges of the traditional facility-based EBP training program model	SP 2.0 clinical telehealth model
Sustaining trained staff enterprise wide. Staff turnover impacts availability of specific EBPs across facilities.	Timely training for new staff. Regional teams that are virtual can provide coverage to large catchment areas of veterans despite staffing fluctuations. Can also utilize cross-VISN partnerships if needed to address temporary staffing shortages.
Volume of trained therapists does not consistently equate to more veterans receiving EBPs (52, 53).	SP 2.0 Clinical Telehealth therapists serve veterans with EBP-SPs as their primary function.
Lack of nationally standardized pathway to consistently refer eligible veterans to targeted EBP treatments.	The 2024 Deputy Under Secretary for Health’s Priority to Action plan utilized a metric to monitor every facility’s referral rates of eligible, consenting clinically appropriate veterans to EBP-SPs.
Implementing EBPs at protocol-consistent time intervals due to scheduling limitations and high demand for access to general mental health.	Program support for optimum EBP-SP implementation consistent with training (e.g., weekly appointments). CRH focused specialty care allows facility-level teams to improve general mental health access.
Tracking and reporting enterprise-wide treatment implementation, effectiveness, and return on investment that directly connects therapist training to veteran outcomes.	Congressional oversight of program evaluation examining therapist EBP-SP workload and veteran outcomes facilitates transparency, including reporting of fiscal responsibility of training and staffing costs.

### Select clinical interventions

As described above, SP 2.0 Clinical Telehealth implemented four EBP-SPs, all recommended by the 2019 CPG (49). The Safety Planning Intervention (SPI) is a one-time 45 to 60 minute evidence-based clinical intervention designed to mitigate suicide risk by providing an individual experiencing suicidality with a written, personalized safety plan that is to be used in the event of a suicidal crisis (54). Stanley et al. (55) administered SPI in emergency departments to over 1,600 patients who were experiencing a suicidal crisis but not requiring hospitalization. They found that completing the SPI with follow up phone contact was associated with 45% fewer suicidal behaviors over 6 months (55). Of note, the CPG recommendation was for “completing a crisis response plan” and the guideline notes that the crisis response plan and the SPI share similar components. The evidence was also reviewed for SPI in the guideline. SPI was implemented versus the crisis response plan given the existing training program and health care system infrastructure (e.g., safety plan form template in the EHR) already available for safety planning in the VA.

Problem-Solving Therapy for Suicide Prevention (PST-SP) is a cognitive-behavioral treatment aimed at preventing or mitigating suicidal crises by improving an individual’s ability to cope with stressful life experiences and problems that contribute to suicidal thoughts and behaviors (56). The manualized treatment protocol is delivered in 6–12 sessions in an individual format. The overarching goal of PST-SP is to reduce suicide risk by teaching adaptive problem-solving strategies and emotion regulation skills and does so by employing an emotion-centered PST approach to address four common barriers to effective problem-solving: cognitive overload, feelings of hopelessness, intense negative emotions, and ineffective problem-solving. There is a strong evidence base regarding the effectiveness of PST for reducing suicide and death ideation and for a variety of other physical, cognitive, and mental health problems across multiple settings, age groups, and diagnoses (57–65). Similar to protocols employed with veterans PST-SP is recommended for individuals who would benefit from a practical, brief, skills-based intervention to facilitate coping with life stressors and problems associated with their suicide risk.

Cognitive Behavioral Therapy for Suicide Prevention (CBT-SP) is a treatment that uses cognitive and behavioral strategies to facilitate a reduction in the likelihood of future suicidal behavior or attempts (66). CBT-SP is a manualized treatment protocol consisting of 12 to 14 sessions, delivered in an individual format. The objectives of CBT-SP are to 1) build a sense of hope, 2) increase awareness of reasons for living, 3) develop alternative ways of thinking and behaving via skill-building, imagery, and rehearsal techniques, and 4) increase coping skills and self-efficacy to manage crises. CBT-SP has been found to significantly reduce risk of suicidal behaviors for up to 24-months following treatment in community and military samples (67–69).

Dialectical Behavior Therapy (DBT) (70, 71) is an evidence-based cognitive behavioral psychotherapy for treating emotional dysregulation and behavioral dyscontrol, including suicidal and self-harm behavior. DBT’s efficacy and effectiveness have been demonstrated in more than 50 randomized controlled trials (72–76), two of which were conducted with veterans (77, 78). DBT is recommended for individuals with repeated suicidal behavior and Borderline Personality Disorder (BPD). DBT is completed in six to twelve months and includes weekly individual therapy, weekly group skills training, weekly therapist consultation team, and as needed phone coaching. The skills taught in DBT include mindfulness, emotion regulation, interpersonal effectiveness, and distress tolerance. Of note, prior to the COVID-19 pandemic, it was not common practice to provide DBT via telehealth. During the pandemic, most DBT teams in the VA transitioned to telehealth. A survey was conducted of DBT teams across VA to allow their experience to inform implementation of DBT in SP 2.0 Clinical Telehealth (79). DBT was implemented first as a telehealth pilot to allow for iterative changes before expanding to other VISNs.

### Participants

Each facility in a VISN can refer eligible, consenting clinically appropriate veterans to their respective VISN CRH. The primary inclusion criterion for patient referral to SP 2.0 Clinical Telehealth is a recent history of suicidal self-directed violence (SSDV), including a suicide attempt or preparatory behavior in the past 12 months. Preparatory behavior may include any acts or preparation towards engaging in SSDV, but before potential for injury has begun (i.e., action beyond the verbalization of a thought, such as buying a gun, collecting pills, writing a suicide note). For DBT, veterans must also have a documented diagnosis of BPD. The program aligned inclusion criteria as closely as reasonable to the populations for which each EBP-SP was recommended in the CPG (e.g., DBT was recommended for those with BPD and SSDV). Of note, an updated CPG was released in May 2024 (80). During SP 2.0 Clinical Telehealth intake, therapists use a shared decision making approach to collaboratively work with veterans to determine the most appropriate treatment.

Therapists participating in SP 2.0 Clinical Telehealth are licensed independent providers hired by the CRHs to provide EBP-SPs. They primarily include psychologists, social workers, and licensed counselors. Historical SSDV and geographical location trend data informed the staffing model for each VISN CRH location. Based on that data, the virtual therapist team sizes range from 3–14 in each VISN, in addition to size-appropriate teams of administrative support (e.g., scheduling assistants).

### Implementation strategies

Given the scope of implementing a national suicide prevention clinical telehealth initiative, a variety of implementation strategies were used, some of which were described in Context. See **Table 2** for a list of implementation strategies used, operational definition, and the targets of each strategy.

**Table 2:** Implementation strategy name, operational definition, and targets of each strategy using Powell et al. (81) naming convention listed in alphabetical order.

Implementation strategy	Operational definition	Targets of each strategy
Access new funding	VA suicide prevention leadership worked with VHA finance and SP 2.0 Clinical Telehealth Initiative funds were used to hire, fund, and train therapists at each CRH.	• VHA finance office • Presidential budget
Assess for readiness and identify barriers and facilitators	Assessment of readiness and identification of implementation barriers and facilitators was conducted in a variety of ways, including 1) conducting the CBT-SP pilot, 2) surveying VA facilities who transitioned to providing DBT via telehealth during COVID-19 (79), 3) analyzing SSDV rates by geographic location to inform staffing model, and 4) use of the Implementation Checklist described below.	• VISN leadership (CMHOs/CRH leadership) • SP 2.0 Clinical program managers • EBP-SP training team leaders • Human Resources
Audit and provide feedback	During the training programs, therapists were assessed for adherence and competence in each EBP-SP and given feedback to improve fidelity. SP 2.0 Clinical program managers and therapists were given feedback on different aspects of the program across fiscal years. This included the percentage of safety plans addressed during the intake session, percentage of intakes referred from the inpatient units, EBP-SP EHR note template usage, and measurement-based care usage.	• Therapists • SP 2.0 Clinical program managers • CRH leadership
Build a coalition	Built partnerships with a variety of stakeholders beyond the SP 2.0 Clinical Telehealth Workgroup; this included partnerships with a variety of other offices (see right), leadership, program evaluation, and suicide prevention and implementation science subject matter experts. Built coalitions within the program. For example, built coalitions among program managers by coordinating “support huddles” of project managers where VISNs with more versus less experience were connected to establish mentor relationships.	• National leadership • CRH leadership • Office of Connected Care • Office of Mental Health (including EBP, Measurement Based Care, and Whole Health programs) • Office of Primary Care • Health and Informatics • Human Resources • National Center for Organizational Development • Program evaluation centers (e.g., Northeast Program Evaluation Center, Program Evaluation Resource Center, Serious Mental Illness Treatment Resource and Evaluation Center, Behavioral Health Quality Enhancement Research Initiative [QUERI]) • Rocky Mountain Mental Illness Research Education and Clinical Center (MIRECC) • Four teams of subject matter experts for each EBP-SP training program
Change physical structure and equipment	Used the VA’s digital divide consult to provide veterans access to internet capable devices.	• Patients • Facility providers • Office of Connected Care
Change record systems	Created EHR note templates for CBT-SP, PST-SP, and DBT to document protocols and reduce burden (while managing how to accommodate the rollout of a new EHR), embedded data elements within EHR note templates to support program evaluation, added measures to Behavioral Health Lab (BHL) (82, 83) Touch software that allows therapists to send assessment measures directly to patients, established a contract with DocuSign for obtaining veteran written consent virtually for audio recording during EBP-SP training, updated the EBP training portal with new EBP-SPs.	• SP 2.0 Clinical therapists • Patients • EBP-SP training program teams • Program evaluation teams • BHL Touch team • DocuSign team • Oracle health team
Change service sites	The goal of this initiative was to bring suicide prevention care to veterans in their home or a private safe location of their choice via telehealth from the CRHs, rather than requiring veterans to drive to a hospital clinic to get services.	• Patients • Office of Connected Care • CRH
Conduct educational meetings	Conducted educational meetings for different audiences throughout VA to encourage referrals of consenting eligible clinically appropriate veterans. In later years of implementation, educational sessions were routinely provided to specific audiences including nursing staff, Suicide Prevention Coordinators, EBP Coordinators, and both outpatient and inpatient mental health providers.	• Referring outpatient providers across VA • Suicide Prevention Coordinators • SP 2.0 Clinical therapists • Advanced Medical Support Assistants • Telehealth Clinical Technicians • CMHOs • EBP Coordinators • Veterans Crisis Line staff • Outpatient and inpatient mental health facility leadership
Conduct ongoing training	Each EBP-SP training program provided 1–3 training opportunities a year. Training cohort size ranged from approximately 2–50 training participants. Training programs typically last between 4–12 months and include independent pre-work, didactic education, clinical demonstrations, experiential role-plays, and ongoing consultation with a small group and expert consultants.	• SP 2.0 Clinical therapists • EBP-SP training program teams
Create new clinical teams	Funded by suicide prevention leadership, each VISN CRH hired new therapists and program managers to provide the EBP-SP and scheduling staff to provide administrative support.	• CRH • Human Resources • Workforce Management • SP 2.0 Clinical therapists • SP 2.0 Clinical program managers
Develop a formal implementation blueprint	Developed an Implementation Checklist for the overall program and one for VISN CRH Teams. The Implementation Checklists were living documents that expanded frequently to accommodate a fast-growing national roll-out across 3 waves of hires, 7 waves of trainings, 2 waves of a global pandemic, and 2 EHRs (see **Appendices A** and **B** for implementation checklists).	• CRH leadership • Facility leadership • SP 2.0 Clinical program managers
Develop academic partnerships	Partnered with academic partners within and external to VA to create gold-standard training programs and expert training program evaluation plans.	• Behavioral Health QUERI • Rocky Mountain MIRECC • Maintaining Implementation through Dynamic Adaptations (MIDAS) QUERI • Non-VA academic partners (e.g., subject matter experts from University of Pennsylvania)
Develop and distribute educational materials	Created marketing and educational materials for national distribution for different audiences named above in ‘conduct educational meetings.’	• Referring providers across VA with a focus on mental health providers
Make training dynamic	Each EBP-SP training team created interactive training experiences that incorporated independent pre-work, didactic seminars, clinical demonstrations, experiential role-play sessions, and ongoing consultation including behavioral rehearsals.	• SP 2.0 Clinical therapists • SP 2.0 Clinical consultants • EBP-SP training program teams
Obtain and use patients/consumers and family feedback	Solicited customer experience data from therapists and veterans to inform data-driven program improvements. For example, conducted annual polls for SP 2.0 Clinical therapists to inform content, frequency, and activities for Community of Practice calls. Facilitated focus groups with therapists to inform changes to the EHR templates. Engaged in focus groups with referring providers, therapists, and CRH leadership during virtual VISN-wide site visits over two years. Examined data for eligible veterans who did not reach intake status and used those data to change the referral template to increase the likelihood of attendance (e.g., confirming phone number and asking for best times to call). Evaluated veteran data to confirm satisfaction with telehealth technology.	• SP 2.0 Clinical therapists • Patients
Provide ongoing consultation	After training participants complete the independent pre-work and didactic seminar portion of the training process, each EBP-SP Team provided ongoing group consultation led by an EBP-SP expert as part of the training program requirements.	• SP 2.0 Clinical therapists • SP 2.0 Clinical consultants
Purposely reexamine the implementation	Monitored progress and adjusted implementation strategies using collaboration with program evaluation team and routinely using dashboard reports to inform program managers about ways to improve program with data driven changes.	• SP 2.0 Clinical program managers • Program evaluation teams
Stage implementation scale up	Phased implementation efforts by starting with telehealth pilots of training programs to assess feasibility and acceptability of virtual EBP-SP treatment. Hired staff in three waves over two years, including approximately six VISNs per wave. Added more staff over time as the program ramped up. Began with goals of nation-wide accessibility (e.g., building the infrastructure such that it was possible for veterans to engage in treatment from every VA health care system in the U.S.) then transitioning to goals of population reach (e.g., monitoring the referral of any eligible consenting clinically appropriate veteran in every catchment area).	• VA suicide prevention leadership • CRH leadership (e.g., Directors, Chief Mental Health Officers)
Use advisory boards and workgroups	Created an initial interdisciplinary SP 2.0 Clinical workgroup from charter to create the program. Additional workgroups were developed such as one for each EBP-SP training program, program evaluation, budget, burnout mitigation, and postvention.	• Leadership (e.g., VA suicide prevention, Office of Connected Care, CRH) • SP 2.0 Clinical therapists

### Measures

We used RE-AIM (84) as our evaluation framework. RE-AIM examines Reach into the target population, Effectiveness of the intervention, Adoption by the setting, Implementation consistency or fidelity, and Maintenance over time. Incorporating stakeholder input, we identified key measures; see **Table 3** for how we defined each RE-AIM dimension. Of note, this paper will not report results for all measures, and will focus on preliminary reach, adoption, and maintenance outcomes. Future reports will include implementation fidelity and effectiveness outcomes.

**Table 3:** RE-AIM dimensions measured with their definition and data source.

Measure	Definition	Data source
Reach	• Number and % of regions and facilities where services are available • Number of veterans referred to SP 2.0 Clinical Telehealth • Program Engagement: Number of veterans with a referral placed and number and % who attended an intake • SP 2.0 Clinical facility-level performance metric: % of patients with suicide behavior event days in the past four quarters that result in a referral being submitted to SP 2.0 Clinical Telehealth • Treatment Engagement: Number of veterans who attended an intake and number and % who started an EBP-SP • Treatment Engagement: Of those who started an EBP-SP, average number of sessions and number and % of treatment completers	Administrative data
Effectiveness	• Suicide-related coping (Suicide Related Coping Scale) (85)^,a^ • Depressive symptoms (PHQ-9)^a^ (51) • Suicide cognitions (Suicide Cognition Scale-Revised) (86)^,b^ • Negative problem orientation (Negative Problem Orientation Questionnaire) (87)^,b^ • Emotion dysregulation (Difficulty in Emotion Regulation Scale-16)^c^ (88) • Symptoms of BPD (Borderline Symptom List)^c^ (89) • Suicide behaviors (e.g., suicide attempts, deaths by suicide) • Inpatient admissions	Administrative data
Adoption	• Number of staff hired/retained • Number and % of hired staff who are currently serving in the program trained in each EBP-SP • Of trained staff currently serving in the program, % of patient encounters where EBP-SP was provided, documented by EBP-SP EHR note templates	Administrative data
Implementation fidelity	• % of Intake Assessments that address the Safety Plan • PST-SP: % of treatment completers who complete all four PST-SP toolkits • CBT-SP: % of treatment completers who complete all three phases of CBT-SP • DBT: % of DBT teams reaching fidelity on the DBT Program Fidelity Measure	Administrative data Survey data
Maintenance	• Ongoing volume of referrals (sustained reach) • Ongoing hiring to address staff turnover (sustained adoption)	Administrative data

a Measure used by PST-SP, CBT-SP, and DBT.

b Measure used by PST-SP only.

c Measure used by DBT only.

Two dashboards collected the data of interest: (1) the EBP Training Data Portal for elements related to tracking therapist training requirements (e.g., adherence and competency to EBP treatment fidelity) and (2) the SP 2.0 Clinical Telehealth Implementation dashboard, which was specifically built to monitor and evaluate the SP 2.0 Clinical Telehealth Program. Monitoring and evaluation were conducted via data collection and visualization tools created to report outcomes consistent with the RE-AIM framework.

The EBP Training Data Portal is an internal web-based tool developed to support all VA EBP training programs and their training participants, consultants, and program administration staff. The main function of the portal is to provide a consistent way to enter and track training cases (e.g., training case demographics, sessions, measurement-based care assessments), track therapist progress toward meeting training requirements, and facilitate discussion for consultation calls. With informed consent, veteran patients may choose to allow their therapy sessions to be audio-recorded as part of their therapist’s training process. Recordings are stored and reviewed in the portal by consultants for adherence and competency evaluation before being deleted. With respect to the RE-AIM framework, the EBP Training Portal focuses most specifically on the elements of Implementation Fidelity.

The SP 2.0 Clinical Telehealth Implementation Dashboard was designed to monitor enterprise-wide outcomes consistent with elements of the RE-AIM framework. For reach, the dashboard monitors the number of veterans referred (and from what facility, what region in the country, and from what kind of treatment setting) as well as the program and treatment engagement as the veteran journeys through the program. For adoption, the dashboard monitors hired staff in alignment with the special purpose funding model and the volume of EBP-SP note templates in the EHR in alignment with the treatment provided. For implementation fidelity, the dashboard monitors treatment completion across the EBP-SPs and examines the intake assessment appointment to notice trends in the safety planning addressed during the first appointment in the program.

The dashboard has been used to provide implementation snapshots on a regular basis; these snapshots are graphical depictions of progress towards the current implementation goal (e.g., hiring, staff trained, volume of referrals). There has been select major implementation foci per year and the snapshots have evolved to showcase the focus. At the beginning of program implementation, the focus was on staff hired and hired staff trained in EBP-SPs. Once the referral consult was accessible at the national level for providers to refer patients, the focus evolved to reach every health care system in the country. To demonstrate that access was available, and the referral process was working, the goal was for each of the 139 VA health care systems^1^ in the U.S. to submit a referral for a patient that is received by a CRH team. Once that goal was achieved, the focus shifted to examining the volume of referrals and the volume of intake appointments (see program engagement in the table above). Finally, the current focus has transitioned to program and treatment engagement, looking beyond the first appointment to continuity of care within therapy protocols.

The implementation snapshots assessed progress on national priorities and served as a messaging strategy to the field. Snapshots presented data by VISN so that all stakeholders could see progress across and within VISNs. VISN leaders could use the data to inform how to promote SP 2.0 Clinical within their VISN. For example, once the snapshots included information about work settings making referrals that attended an intake appointment (e.g., primary care mental health integration, residential care), program managers could do localized targeted messaging to increase referrals.

A SP 2.0 Clinical facility-level performance metric was established in FY23 to monitor and incentivize every facility to refer eligible consenting clinically appropriate veterans to the program. This was also displayed on the dashboard. The metric consists of the percentage of patients with suicide behavior event days in the past four quarters that resulted in a referral to SP 2.0 Clinical Telehealth. A suicide behavior event day is defined as a unique day within a facility in which a patient reported one or more suicide behaviors, including preparatory behavior. This metric was highlighted by the Under Secretary for Health’s Priority to Action initiative (90), which set facility level goals for expected improvement in FY24.

### Analysis

Summary statistics of the reach, adoption and associated maintenance measures under the RE-AIM framework were conducted to assess implementation using VA administrative data and analyzed with SAS 9.4. Unless otherwise noted, data from April 1, 2021 through September 30, 2024, representing three and a half years of implementation. Except for reach data (number of VISNs and health care systems, number of referrals) and therapist data (number hired, percent retained, and training completion data), administrative data were available from 134 (of 139) health care systems due to transition to a new EHR during the project period.

## Results

### Reach

**Figure 3:**
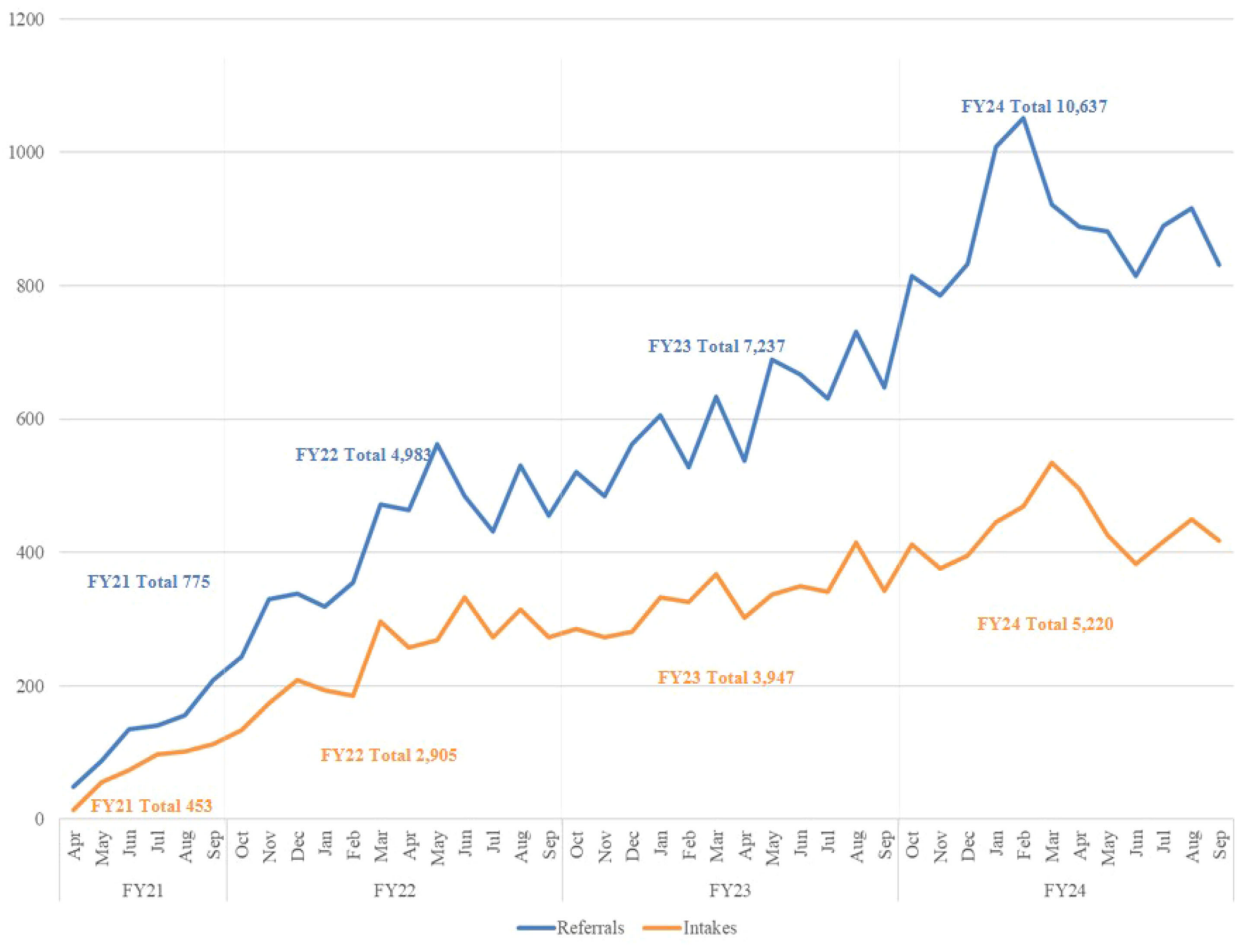
Number of SP 2.0 Clinical Telehealth referrals and intakes from April 2021 through September 2024. *Based on timing of program implementation start date, FY21 does not represent an entire fiscal year.

By April 2023, SP 2.0 Clinical Telehealth services were available in 18 of 18 (100%) VISNs and in 139 of 139 (100%) health care systems in the U.S. By the end of September 2024, 23,632 patients were referred for care. See **Figure 3** for the number of referrals and intakes each month and the totals per fiscal year (FY).

**Figure 4:**
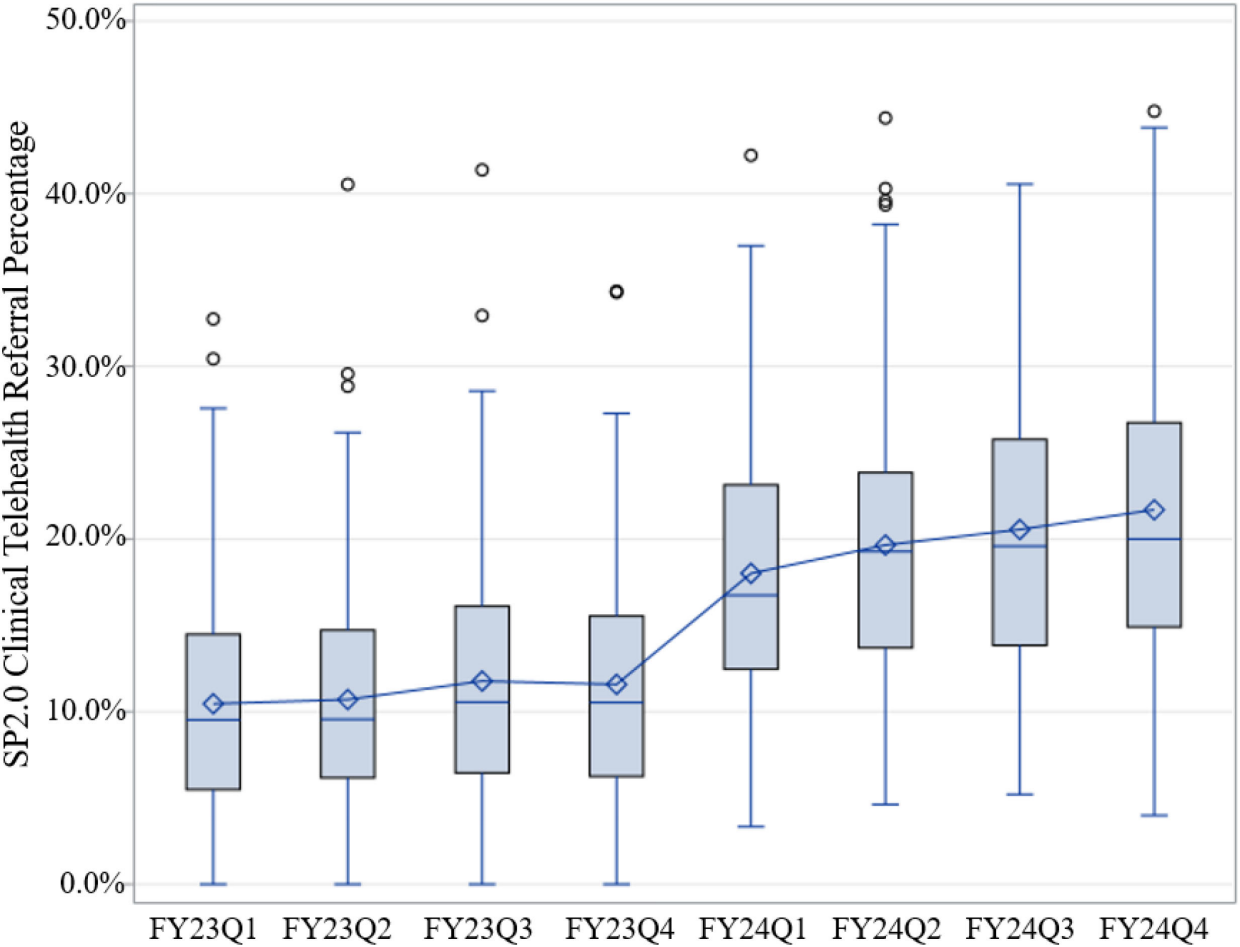
Measures of dispersion (mean, median, interquartile range, minimum and maximum values, and outliers) of SP 2.0 Clinical Telehealth referral percentages by FY and quarter.

Regarding the SP 2.0 Clinical facility-level performance metric that examined the percentage of patients with suicide behavior event days that resulted in a referral to SP 2.0 Clinical Telehealth, the mean percentage of eligible patients receiving referrals showed an increase from 10.5% in the first quarter of FY23 to 21.7% in the fourth quarter of FY24 (see **Figure 4**).

To examine program engagement, we reviewed referrals received through the end of September 2024. Of the 23,632 referrals placed, 12,701 (53.75%) resulted in a completed intake. Of the completed intakes, 11,960 (94.17%) received further intervention, defined as a safety plan either created, updated, or reviewed and/or attended one or more EBP-SP session.

Tracking of the provision of the full safety planning intervention began on July 1, 2023. Of the 6,318 intake appointments that occurred from July 1, 2023 to September 30, 2024, providers addressed safety plans in 5,815 (92.04%). This included reviewing an existing safety plan (4,471; 76.89%) with no changes, updating an existing safety plan (792; 13.62%), and completing a new safety plan (504; 8.67%). Among those who developed a new safety plan, 55.95% (282) were developed in the context of completion of the full SPI.

For treatment engagement, we examined the number of patients who attended an intake and completed at least one session of an EBP-SP psychotherapy. Of those who completed an intake (12,701), 8,758 (68.96%) completed at least one session. Among patients who received at least one session, 36.07% received PST-SP only, 48.80% received CBT-SP only, 4.22% received DBT only, and 10.90% received more than one type of treatment. Of those who initiated treatment, 4,222 (48.21%) completed a full protocol of treatment by the close of June 30, 2025, as denoted by their therapist in a discharge EHR note template. Among patients who completed treatment by the end of September, 2024 (8,363) received an average of 6.26 sessions of PST-SP (n=2,856) and 8.86 sessions of CBT-SP (n=3,747) (DBT data still pending), indicating that a significant percentage of patients reached at least the middle stages of treatment.

### Adoption

As of September 30, 2024, the program hired 137 therapists and retained 107 (78.10%) in their role within SP 2.0. Of those 107 retained therapists, at the close of September 2024, 100% were trained in two or more EBP-SPs, 91.59% were trained in three or more EBP-SPs, and 42.99% were trained in all four EBP-SPs. We define trained as 1) completed all pre-requisites for a training, 2) completed workshop portion that includes didactics and experiential role plays, and 3) currently seeing cases and in weekly consultation or having a certificate of completion. Further, in FY24 (October 1, 2023-September 30, 2024), 32,946 (70.08%) of clinical appointments within the SP 2.0 Clinical Telehealth program (N = 47,012) included provision of EBP-SP psychotherapy (PST-SP, CBT-SP, DBT) based on use of standardized EHR note templates.

### Maintenance

**Figure 5:**
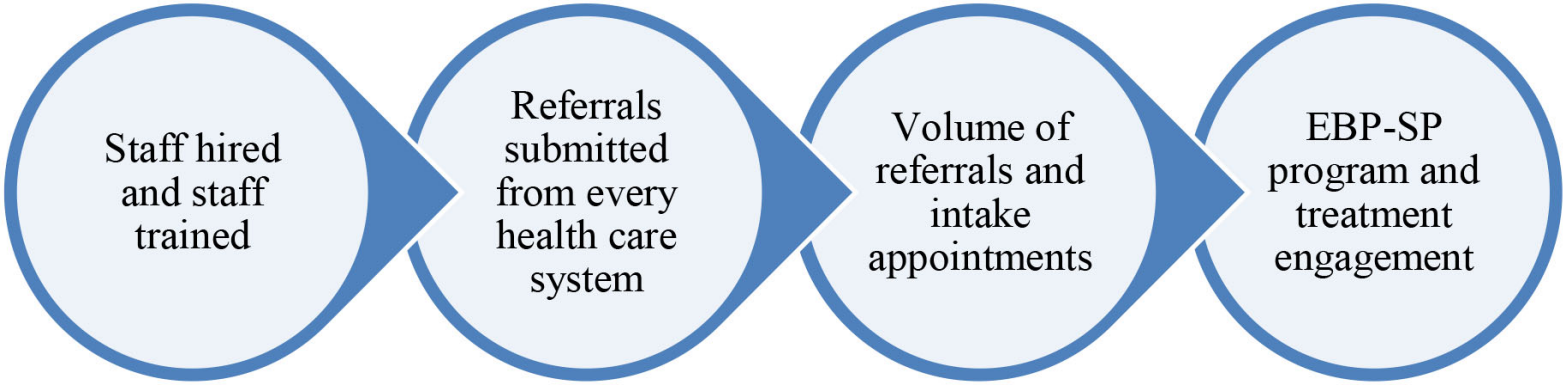
Implementation foci over time.

By the end of FY24 the program received 23,632 referrals nationwide. Increasing referral rates (see **Figure 5**) year over year (45.23% increase from FY22-23, 46.92% increase from FY23-24) suggests ongoing sustained reach (Maintenance). By the close of FY22, SP 2.0 Clinical Telehealth teams had reached 89.57% staffing of the number of recommended therapist positions. Even with the percent of approved staff positions increasing by 1.74% from FY22-24, staffing levels increased to 91.45% in FY24, demonstrating the program’s ability to manage turnover over time and sustained adoption.

## Discussion

The SP 2.0 Clinical Telehealth program is the first and only national, enterprise-wide, fully virtual clinical infrastructure creating access for veterans to receive evidence-based suicide prevention clinical treatment. The program addressed challenges to VA’s traditional EBP training model, as well as known barriers to care for veterans, by providing care nationally via telehealth using the existing CRH structure. The program used a host of implementation strategies and engaged stakeholders at all levels of VA to put this portion of the national strategy for suicide prevention into practice.

The program’s implementation was successful in reaching all VISNs and all VA health care systems in the U.S. In the first three and a half years, over 23,000 patients were referred for care and a notable proportion of those referred were successfully evaluated for and engaged in treatment. Over half of those referred attended an intake appointment and 94.16% of individuals attending the intake received further intervention (safety plan addressed and/or EBP-SP). For patients who completed an intake, 68.96% engaged in treatment by completing at least one session of an EBP-SP psychotherapy and of those, 48.21% completed a full course of treatment.

Patients who completed PST-SP and CBT-SP reached the middle to late stages of treatment, receiving an average of 6.26 and 8.86 sessions respectively. Importantly, this “real-world” length of care is comparable to length of treatment received in several randomized controlled trials (RCT) for these treatments that demonstrate clinical improvement. For example, in a RCT of PST that compared in-person PST, PST via telehealth (tele-PST), and telephone support (control condition), PST was offered for 6 sessions. Results indicated that the impact of tele-PST on depression and disability outcomes were sustained significantly longer than in-person PST (91). They also found that tele-PST (but not in-person PST) was more effective than the control in reducing death/suicidal ideation (92). In one RCT of CBT-SP patients received an average of 8.92 (SD = 5.97) sessions. Those who received CBT-SP versus control had a significantly better outcomes (i.e., lower suicide reattempt rate, lower self-reported depression, and less hopelessness) (67). Therefore, results regarding number of EBP-SP psychotherapy sessions completed are promising. Evaluation is ongoing to determine the impact of that dose of therapy on patient outcomes in SP 2.0 Clinical Telehealth.

In terms of adoption, most SP 2.0 Clinical Telehealth therapists have been trained in three of the four EBP-SPs. Further, despite a slower DBT implementation to allow for iterative change to the training program based on telehealth pilot results, over 40% of therapists have been trained in all four EBP-SPs. Once trained, available data also confirms that therapists are dedicating their clinical time to provision of approved EBP-SPs as intended, with 70.08% of clinical appointments documented using EBP-SP EHR note templates.

There is evidence of sustained reach and adoption when examining the maintenance of implementation of the SP 2.0 Clinical Telehealth Initiative. Sustained reach was demonstrated with increasing referral rates over the years of program implementation. A notable contributor to this sustainment was the establishment of the SP 2.0 Clinical Telehealth facility-level performance metric, which demonstrated an increase in the percentage of potentially eligible patients referred nationally each quarter. Implementation of this metric allowed VA suicide prevention leadership to successfully monitor and measure an EBP-SP referral system (at the enterprise level) that held VA health care systems accountable (at the facility level) focusing on veteran care (at the patient level). Finally, the program reached 89.57% staffing, and that number slowly increased over the next two fiscal years. Despite some turnover, the program was able to sustain adoption and increase staffing.

Several factors supported VA’s success in this implementation. First, during program development, VA suicide prevention leadership engaged stakeholders at all levels of VA and VHA finance to support this program’s approval and funding. Second, the SP 2.0 Clinical Telehealth program hired over 100 psychotherapists with teleworking agreements and tours of duty that allowed for provision of care across multiple time zones. As federal employees working for VA, the Supremacy Clause 38 C.F.R § 17.419 (2025) and the Anywhere to Anywhere regulation 38 C.F.R. § 17 (2017) allows mental health providers licensed in one state to serve veterans in other states. Therefore, VA could cast a wide net to hire the most competitive applicants without limiting the search to geographic restrictions or requiring applicants to move. Third, once hired, all therapists were embedded into a CRH infrastructure, made up of regionally-based telehealth teams, allowing for nationwide veteran reach of this fully virtual model. Fourth, the therapists received gold standard, competency-based training in EBP-SPs based on recommendations from the 2019 CPG for the assessment and management of patients at risk for suicide. Training programs were piloted, and iterative changes were made to improve therapists’ experience and training outcomes.

Finally, the development and utilization of multiple dashboard reports allowed VA suicide prevention leadership to monitor and provide feedback on the reach, effectiveness, adoption, implementation, and maintenance of this program, driving continuous quality improvement. The dashboard reports also evolved to focus on program engagement and treatment engagement. This allowed for examination of referrals, intakes, percentage of veterans consenting to come back and engage in more EBP-SP and ultimately look at percentage of treatment completion.

### Next steps

VA suicide prevention leadership continues its focus on program improvement. For example, EBP-SP training programs are assessing and adapting the training programs based on training participant progress and feedback and site visits offer the opportunity to identify other areas for improvement. The program was implemented based on recommendations from the 2019 CPG. A revised CPG was published in 2024, and the update included changes in methodology and focus that resulted in changes in the ratings of evidence-based practices since the 2019 version. Like with the updated CPG, VA suicide prevention leadership is continually assessing how the program can evolve in response to new research as it becomes available.

As the program continues and serves more veterans, additional data will become available. Regarding effectiveness, evaluation of patient outcomes (i.e., changes in symptoms as demonstrated by standardized measures) is ongoing. Evaluation of self-directed violence and suicide behaviors including death by suicide are planned. Each training program is evaluating the impact of their respective trainings on therapist adherence and on implementation fidelity.

### Implications

Implementation of the SP 2.0 Clinical Telehealth program demonstrated how strategic use of implementation science strategies supported successful implementation of a new national approach to providing evidence-based practices for suicide prevention via telehealth to veterans across the U.S. These implementation science strategies included engaging leadership across multiple levels of the health care system, accessing new dedicated suicide prevention funding, and engaging implementation science experts and program evaluation partners from the onset. Telehealth was thoughtfully selected as a treatment modality to enhance reach to veterans, especially for those in rural areas (93). Telework was used for hiring to attract the most qualified applicants regardless of geographic region. The partnership with a telehealth infrastructure (CRH) facilitated enterprise-wide reach and hiring of therapists. Training programs were developed to provide appropriate training and support to therapists and their supervisory chain of command, so the entire team had the tools and skills to navigate implementation steps. This all resulted in improved access to evidence-based suicide prevention clinical care for veterans within VA. The SP 2.0 Clinical Telehealth program can be used as a model for other large health care systems looking to improve provision of evidence-based interventions for suicide prevention.

## Conclusion

SP 2.0 Clinical Telehealth represents the first and only enterprise-wide fully virtual evidence-based treatment program for veterans with a recent history of suicidal self-directed violence. The model uses a fully virtual telehealth team-based approach, addressing access barriers of face-to-face care (e.g., transportation time and cost, childcare, limited clinic hours). Program funding is specific purpose funded to support the SP 2.0 Initiative, as outlined within the VHA Presidential budget. As such, therapists are fully dedicated to the program and have supervisory support to engage in EBP-SPs. This gives SP 2.0 Clinical Telehealth therapists the time and tools they need to provide EBP-SPs according to their respective protocols. Notably, perceived institutional support for providing EBP has been linked to less burnout and more job satisfaction for VA providers (24). Within two years, all VISNs and all VA health care systems in the U.S. had access to these services. In the first three and a half years, over 23,000 veterans were referred to care and over half completed an intake. Almost all of those who completed an intake had a safety plan completed, updated, or reviewed, in the first clinical contact. After intake, 68.96% completed at least one session of an EBP-SP psychotherapy and nearly half of those patients completed a full course of treatment. The program continues to evaluate outcomes and make iterative changes to improve suicide prevention intervention for veterans.

## Data Availability

The datasets presented in this article are not readily available because they include PHI from VA healthcare systems. Requests to access the datasets should be directed to the corresponding author.

## Footnotes

1 VA’s 170 medical centers and 1,193 outpatient sites described in Setting are housed within 140 health care systems, 139 are within the U.S. and one is in Manilla, Philippines. VA is not allowed to provide telehealth internationally.
