# Supplementary material for: Implementation of a fully virtual enterprise-wide clinical evidence-based suicide prevention program in the U. S. Department of Veterans Affairs: the suicide prevention 2.0 clinical telehealth initiative
Source: Front Psychiatry. 2026 Feb 10;16:1668417. doi: 10.3389/fpsyt.2025.1668417 (PMC12930348; doi:10.3389/fpsyt.2025.1668417)
Supplement: Supplementary file 1 [file SupplementaryFile1.docx]

**Appendix A: Implementation Checklist for Overall SP 2.0 Clinical Telehealth Program**

| **STRATEGY** | **Overall Status** | **Details** | **Next Big Priority/ Challenge/ Deliverable** | **Data As Applicable** |
| --- | --- | --- | --- | --- |
| Strategy 1a: Establish partnerships with CRH leadership at national and VISN Levels |  |  |  |  |
| Strategy 1b: Ongoing partnership with CRH (includes ongoing communication infrastructure & streams) |  |  |  |  |
| Strategy 2: Implement hiring and training timeline |  |  |  |  |
| Strategy 3: Establish and implement a national virtual training program for CBT-SP |  |  |  |  |
| Strategy 4: Collaborate with EBP program office for partnership with the Advanced Training of the Safety Planning Intervention (ASPI) program |  |  |  |  |
| Strategy 5: Collaborate with EBP program office to establish and implement a national virtual training program for PST-SP |  |  |  |  |
| Strategy 6: Collaborate with BH QUERI to establish and implement a pilot virtual DBT training program with four VISN CRHs |  |  |  |  |
| Strategy 7: Establish infrastructure for clinical flow of the veteran customer experience; develop training for key stakeholders including the roles and responsibilities of the referring provider, suicide prevention telehealth providers, and administrative staff |  |  |  |  |
| Strategy 7a: Referring Providers/Facility Mental Health Point of Contact |  |  |  |  |
| Strategy 7b: Therapists |  |  |  |  |
| Strategy 7c: Admin/Advanced Medical Support Assistants |  |  |  |  |
| Strategy 8: Create and implement clinical operational tools including an interfacility consult, a consult response note, EBP EHR note templates, and a discharge template |  |  |  |  |
| Strategy 9a: Evaluation of Training Program (Training Participants’ Outcomes) |  |  |  |  |
| Strategy 9b: Develop and deploy program evaluation plan (Implementation Program Evaluation) |  |  |  |  |
| Strategy 10: Develop and disseminate a marketing/ communications plan |  |  |  |  |
| Strategy 11: Establish suicide prevention telehealth providers’ schedule of ongoing consultation and community of practice calls |  |  |  |  |
| Strategy 12: Establish guidance for provider mapping and workload |  |  |  |  |
| Strategy 13: Develop and disseminate recommendations for burnout mitigation |  |  |  |  |
| Strategy 14: Develop and disseminate recommendations for postvention |  |  |  |  |
