# Supplementary material for: Implementation of a fully virtual enterprise-wide clinical evidence-based suicide prevention program in the U. S. Department of Veterans Affairs: the suicide prevention 2.0 clinical telehealth initiative
Source: Front Psychiatry. 2026 Feb 10;16:1668417. doi: 10.3389/fpsyt.2025.1668417 (PMC12930348; doi:10.3389/fpsyt.2025.1668417)
Supplement: Supplementary file 2 [file SupplementaryFile2.docx]

**Appendix B: Implementation Checklist for VISN CRH Teams Used During Program Launch**

| **PREPARING FOR SP 2.0 CLINICAL TELEHEALTH IN YOUR CRH - TASKS/STEPS FOR IMPLEMENTATION** | |
| --- | --- |
| ***ESTIMATED TIMEFRAMES*** | ***CRH Operations Guide*** |
| FIRST STEP - establish | Telehealth Service Agreements in place |
| FIRST STEP - establish | Local processes/standard operating procedures (SOP) in place for asynchronous measurement-based care (should include timeframes for review and surrogate coverage) |
| FIRST STEP - establish | Emergency procedures established and understood |
| AT ONBOARD - staff aware | Emergency procedures for:  Technological problems  Medical Emergency  Disruptive Behavior  Involuntary Commitment Procedures - know the laws in your state(s) - civil commitment laws and standards that determine eligibility for intervention where the individual in crisis lives.  Child and Elder Duty to Warn/Protect regulations/process in your state(s) |
| AT ONBOARD | Providers are VA Video Connect (VVC; VA’s secure videoconferencing app) ready |
| AT ONBOARD | Provider has government furnished equipment including laptops, phones, and external microphones if needed |
| AT ONBOARD | Clinic Set-Up/Naming Conventions - reminder Intake Appointment needs to be a 90-minute slot |
| ONE MONTH BEFORE READY TO TAKE REFERRALS | Outreach and Marketing Expectations |
| TWO WEEKS BEFORE READY TO TAKE REFERRALS - AFTER STAFF ONBOARD AND TRAINED - Dialogues with Informatics should start far in advance | Work with Informatics Team to activate the consult (decisions should already be made about when and where, based on number of staff onboarded and trained - you do not need to activate at all locations at once) |
| TWO WEEKS BEFORE READY TO TAKE REFERRALS (or earlier) | Names provided to SP 2.0 Clinical Telehealth for two administrative points of contact (POC) (Advanced Medical Support Assistants are ideal) |
| TWO WEEKS BEFORE READY TO TAKE REFERRALS (or earlier) | POCs for clinical emergencies |
| BEFORE CONSULT IS LIVE | Intake Assessment - trained in use, decisions made about who will do intakes |
| BEFORE CONSULT IS LIVE | Local guidelines |
| BEFORE CONSULT IS LIVE | Mental health POCs from referring sites - who are they? |
| BEFORE CONSULT IS LIVE | Communication between SP 2.0 Clinical Telehealth providers and Mental health POC - when and how? During and after treatment? |
| BEFORE CONSULT IS LIVE | Consult Management System - who gets alerted to the consult? Who responds? Who does intakes? Please note Office of Veterans Access to Care Guidelines/VHA Directives on Consult Management |
| BEFORE CONSULT IS LIVE | Technology - test calls - who does them and how do you connect the veteran to technology assistance? |
| BEFORE CONSULT IS LIVE | Mailing audio recording consent forms and collecting wet signatures* for patients who agree to serve as training cases for therapists in EBP-SP training programs |
| BEFORE CONSULT IS LIVE | Waiting for written consent forms* |
| AFTER ONBOARD | VA TRAININGS (via computerized Talent Management System (TMS)):   - Shared Decision Making - Skills Training for Evaluation and Management of Suicide - Lethal Means Safety Counseling to Reduce Suicide Risk - Why Suicide Risk Assessment Still Matters - Suicide Risk Screening and Assessment - Suicide Risk Screening and Assessment Process - Comprehensive Suicide Risk Evaluation - Using Chain Analysis to Assess and Intervene on Suicidal Ideation and Behavior - Welfare Checks and Therapeutic Risk Management - PST-SP Video series and web course (before PST-SP Training) |
| AFTER ONBOARD | EBP Orientation |
| AFTER ONBOARD | EBP Workshop |
| AFTER ONBOARD | EBP Consultation |
| ONGOING | SP 2.0 Clinical Telehealth Flow - use this document as a reference to demonstrate ~~the~~ patient flow ~~in SP 2.0~~ |
| ANYTIME PRIOR TO PATIENT CARE | Names provided to SP 2.0 Clinical Telehealth for access to data reports (Power BI) |
| If your team is providing DBT | For CRHs with DBT providers - Group Telehealth Agreement (GTA) |

*Note: As described in the main text of the manuscript, a solution was later put in place to collect signatures virtually via DocuSign.
